# Infant HIV Testing Amid the COVID-19 Pandemic and Evolving PMTCT Guidelines in Johannesburg, South Africa

**DOI:** 10.3390/tropicalmed7100302

**Published:** 2022-10-15

**Authors:** Coceka N. Mnyani, Andomei Smit, Gayle G. Sherman

**Affiliations:** 1 Department of Obstetrics and Gynaecology, School of Clinical Medicine, University of the Witwatersrand, Johannesburg 2193, South Africa; 2 Centre for HIV and STIs, National Institute for Communicable Diseases, Division of the National Health Laboratory Service, Johannesburg 2192, South Africa; 3 Department of Paediatrics and Child Health, School of Clinical Medicine, University of the Witwatersrand, Johannesburg 2193, South Africa

**Keywords:** PMTCT, HIV, COVID-19

## Abstract

**Background:** The COVID-19 pandemic impacted HIV programmes with the diversion of resources and lockdown measures. We assessed the impact of COVID-19 on infant HIV diagnosis in the context of updated 2019 prevention of mother-to-child transmission of HIV (PMTCT) guidelines in Johannesburg, South Africa. **Methods:** HIV PCR data for children <2 years were extracted from the National Health Laboratory Service database from October 2018 to September 2021, inclusive. Trends in the total number of tests performed and the total number of children with HIV diagnosed, stratified by age, were determined to assess the effect of different COVID-19 lockdown levels and updated guidelines. **Results:** When comparing three 12-month periods ending September 2019–2021, respectively, the total number of HIV PCR tests performed increased (from 41 879 to 47 265 to 56 813), and the total number of children with HIV decreased (from 659 to 640 to 620), year-on-year. There was a substantial increase in 6-month testing in response to updated guidelines. Excluding 6-month testing, the year-on-year increase in total tests was maintained with birth and 10-week testing closely approximating total live births to women living with HIV. A decrease in the total number of children with HIV diagnosed was noted in Q2 2020, coinciding with the most restrictive lockdown, followed by a rebound in cases. **Conclusions:** Despite the restrictions and diversion of resources associated with COVID-19, there was a successful implementation of PMTCT guideline updates and minimal disruption to infant HIV testing. However, much work remains in order to achieve the elimination of mother-to-child transmission of HIV.

## 1. Introduction

COVID-19, which was declared a pandemic in March 2020, has had a devastating impact globally, not only in terms of morbidity and mortality associated with infections but also impact on health programmes, including the provision of HIV care. Various commentaries and modelling studies predicted the negative impact the pandemic would have on HIV programmes, as resources were diverted and various lockdown measures implemented in a bid to curb the spread of infections [1,2,3,4]. In a model-based analysis of the potential impact of COVID-19-associated interruptions in the UNAIDS 21 priority countries, there were projected interruptions in paediatric HIV prevention and care [4]. The projections included a decline in pregnant women accessing prevention of mother-to-child transmission of HIV (PMTCT) services, with a subsequent increase in new paediatric HIV infections and disruptions in care for children living with HIV. The analysis could not project the impact on infant HIV testing as a result of insufficient pre-COVID-19 data from the countries [4].

The published data have subsequently shown the impact of COVID-19 throughout the HIV care cascade, and the impact is likely to be felt for years to come. Early in the pandemic, the Global Fund reported that several countries reported high or very high-level disruptions in HIV care, with the prevention, testing and support of people living with HIV the most impacted [5]. South Africa, where the first case of COVID-19 was identified on 5 March 2020, and a stringent national lockdown subsequently implemented on 27 March 2020, has not been spared from the effects of the pandemic [6]. At the time, the country was implementing revised PMTCT guidelines, which included the introduction of dolutegravir-based antiretroviral therapy (ART) and changes in maternal monitoring and infant HIV testing [7]. A July 2020 UNICEF report showed limited implementation of the guidelines [8]. Hence, the aim of this study was to assess the impact of COVID-19 on infant HIV diagnosis in the context of updated 2019 PMTCT guidelines in the Johannesburg Health District, South Africa. 

## 2. Materials and Methods

### 2.1. Study Setting

The Johannesburg Health District has seven subdistricts, A–G, and had a population of over five million people in 2021 [9]. The HIV prevalence in pregnant women in the district was estimated to be around 25% in 2019, with high ART coverage of over 90% during pregnancy [10]. The revised PMTCT guidelines were signed off for implementation in November 2019. The revisions included the introduction of dolutegravir-based ART for first-line treatment, initially with caution for use in the first trimester because of the then-reported increased risk of neural tube defects with periconception use [11,12]. This caution was later removed in July 2021 from the South African PMTCT guidelines as accumulating data showed no increased risk of neural tube defects with dolutegravir use compared to other ART regimens [13]. 

Several updates related to routine infant HIV testing were made [7]. In addition to the birth and 10-weeks HIV PCR testing that was already in place, a 6-months PCR test was added for all HIV-exposed infants who had previously tested HIV-negative. The rationale for choosing the 6-month instead of the 9-month screening recommended by the World Health Organization (WHO) was based on the decreased testing coverage with increasing infant age experienced in early infant diagnosis (EID). Moreover, the findings from a nationally representative South African cohort demonstrated that 82% of cumulative MTCT occurred by 6 months of age, at which time breastfeeding prevalence is less than 30% [14]. Universal HIV testing at 18 months for all infants was also introduced, with a rapid HIV antibody test used for screening and a PCR test used for confirmation of a positive result. Confirmatory PCR testing was recommended for up to two years of age. Age-appropriate HIV testing for symptoms consistent with HIV infection and 6 weeks post cessation of breastfeeding remained in place in the revised guidelines [7]. 

### 2.2. Study Design

HIV PCR data for children aged less than two years who were tested in the Johannesburg Health District in the period 01 October 2018 to 30 September 2021 were extracted from the Surveillance Data Warehouse (DW) at the National Institute for Communicable Diseases (NICD), a division of the National Health Laboratory Service (NHLS). NHLS performs all pathology tests for the public health sector nationally, serving more than 80% of the South African population, and captures these records in a laboratory information system. In order to facilitate the monitoring of HIV programs such as EID of HIV, test data are transferred to the NICD DW. In the absence of a unique health identifier, the DW utilises a probabilistic linking algorithm to link multiple HIV tests belonging to the same patient, employing predominantly the variables name, surname and date of birth to allocate a DW unique identifier. The accuracy of this algorithm is compromised by the use of poor demographic details provided at the time of birth testing that is updated for testing at later time points, precluding accurate longitudinal monitoring from birth [15]. However, where infants test HIV PCR positive, and a confirmatory test follows shortly thereafter, similar demographic details generally allow for linkage facilitating de-duplication of HIV PCR positive tests to infants with HIV. Other variables used include the facility where the specimen was collected, facility GPS coordinates, date of specimen registration and age at testing, HIV PCR test result and the DW unique identifier. 

Data from the DW were compared to EID indicators collected by the District Health Information System (DHIS), which reports on birth and around 10-week HIV PCR tests by counting the total number of tests performed and the total number of positive tests in these two age groups, respectively. DHIS is the South African Department of Health system used to collect aggregate routine data from public healthcare facilities. Birth HIV PCR test is defined by DHIS as tests performed from birth to less than 6 weeks of infant age, and 10-week HIV PCR test as tests performed from 6 weeks to less than 14 weeks of age. As there was no 6-month testing reported on DHIS at the time of the study, the NICD definition of a 6-month HIV PCR test was used and refers to testing performed from 14 weeks of infant age to less than 9 months. Other DHIS indicators used included total live births and total live births to women living with HIV (WLHIV).

### 2.3. Data Analysis 

An analysis was performed using RStudio Version 1.3.1056 on a 64-bit Windows device to generate the total number of tests performed, the total number of children with HIV diagnosed and the geospatial maps. Trends in the total number of tests performed and the total number of HIV PCR-positive tests, stratified by age, were determined to assess the effects of the introduction of revised PMTCT guidelines and different COVID-19 lockdown levels. Testing coverage was calculated as the total number of HIV PCR tests performed divided by the total number of live births to WLHIV reported by DHIS. Case rates were calculated using the total number of first HIV PCR-positive tests at an age less than 2 years as a proxy for newly diagnosed children with HIV, divided by the DHIS indicator of total live births and expressed per 100,000. In the study, a year was defined as the period from the beginning of the fourth quarter, i.e., 01 October to 31 December, to the end of the third quarter, i.e., 01 July to 30 September. There were five COVID-19 lockdown levels that were introduced at various times in South Africa, with level 5 being the most restrictive, severely limiting people’s movements and economic activity [6]. Healthcare facilities remained open throughout the different lockdown levels. 

### 2.4. Ethics 

Ethics approval for the study was obtained from the University of the Witwatersrand Human Research Ethics committee (Protocol numbers M201186 and M210752). De-identified data were analysed on a secure share point folder hosted by the NICD.

## 3. Results

**Figure 1:**
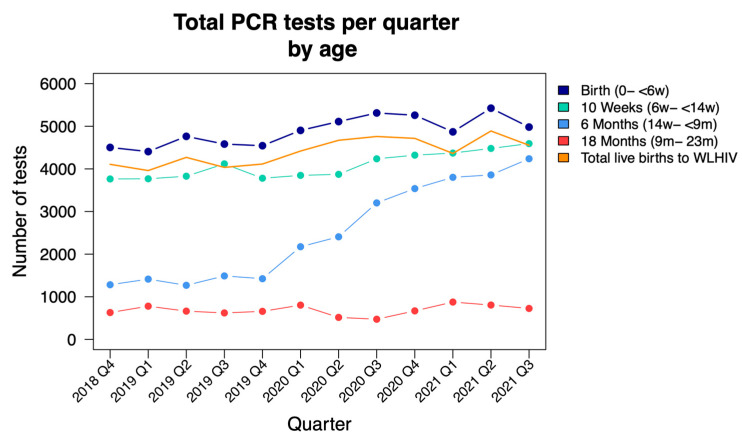
HIV PCR tests performed quarterly from October 2018 to September 2021, stratified by age; NICD data (d = days; w = weeks; m = months; WLHIV = women living with HIV).

A total of 153,723 HIV PCR tests were analysed, belonging to 127,515 unique patients as allocated by the probabilistic linking algorithm in the NICD DW. Total HIV PCR tests performed increased over the study period from 10,179 to 14,538 per quarter, attributable to an increase in tests performed in all age groups but predominantly a consequence of a three-fold increase in testing around 6 months of age in line with new PMTCT guideline recommendations (Figure 1). 

* Total birth tests performed are higher than the total live births to WLHIV, likely due to a combination of under-reporting of the DHIS indicator and repeat HIV PCR tests performed in the first 6 weeks of life.

**Figure 2:**
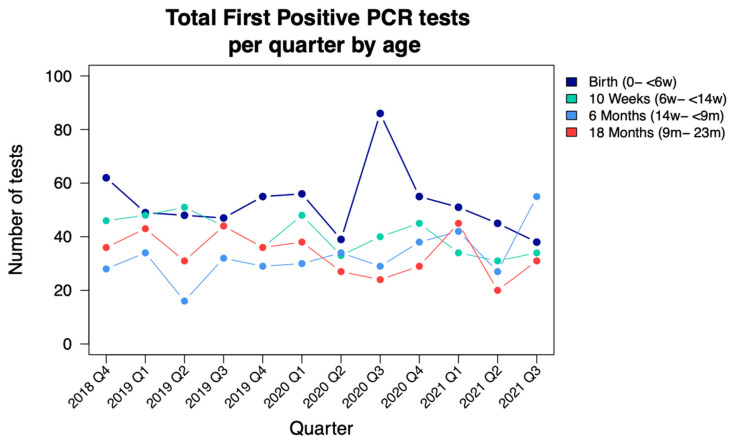
Children testing HIV PCR positive performed quarterly from October 2018 to September 2021, stratified by age; NICD data (d = days; w = weeks; m = months).

Deduplicated HIV PCR-positive test results decreased year on year from 659 to 620, with a total of 1919 newly diagnosed infants and children with HIV identified during the study period (Figure 2; Table 1).

According to the DHIS data, there was a total of 51,334 live births to WLHIV during the study period, with an average of 4278 live births to WLHIV per quarter. The decrease in positive HIV PCR tests translates to a declining annual case rate of newly diagnosed less than two-year-old infants and children of 905 to 804 per 100,000 live births. During the most stringent COVID-19 lockdown period viz. Q2 2020, there appears to be a decrease in the number of newly diagnosed children with HIV at birth and around 10 weeks of age, followed by a rebound when restrictions were eased in Q3 2020. A decrease in total PCR testing at these time periods is less obvious.

Birth testing coverage remained around 100% throughout the study period whilst 10-week testing coverage averaged 85%, with a mild decrease to 79.5% during Q2 and Q3 of 2020. Coverage of 6-month testing increased steadily from 36% to 90% during the study period. Birth testing was the time point that yielded the highest number of first HIV PCR-positive tests in comparison to 10-week and 6-month testing, with a total of 631 cases during the study period in comparison to 490 at 10 weeks and 394 at 6 months. A total of 1515/1919 (79%) first HIV PCR-positive tests were detected in the first eight months of life. The increased tests performed at 6 months of age demonstrated an annual increase in first HIV PCR-positive cases from 110 to 122 to 162, year-on-year. 

During the study period, the total number of HIV PCR tests performed correlated fairly well between DHIS and NICD data—Table 1. Total birth and 10-week tests were recorded by DHIS as 51,815 and 50,335 in comparison to NICD 58,656 and 48,982, respectively. Thus, NICD recorded 5488 more PCR tests being performed at birth and 10 weeks of age. Triangulation of first HIV PCR-positive tests correlates less well with newly diagnosed children at birth and 10-week tests reported by DHIS as 326 and 264 in comparison to NICD 631 and 490, respectively. DHIS reports a total of 590 newly diagnosed infants to 1121 reported by NICD. In order to reduce the effect of NICD data overcounting newly diagnosed infants because of the inability to link patient tests accurately, the age ranges for birth and 10-week testing for NICD data were narrowed from 0–<6 weeks to <7 days of age and from 6 weeks–<14 weeks to 8–12 weeks of age, respectively, which again yielded higher numbers of infected infants than DHIS reports at 441 and 341 newly diagnosed infants at birth and 10 weeks, respectively. 

**Figure 3:**
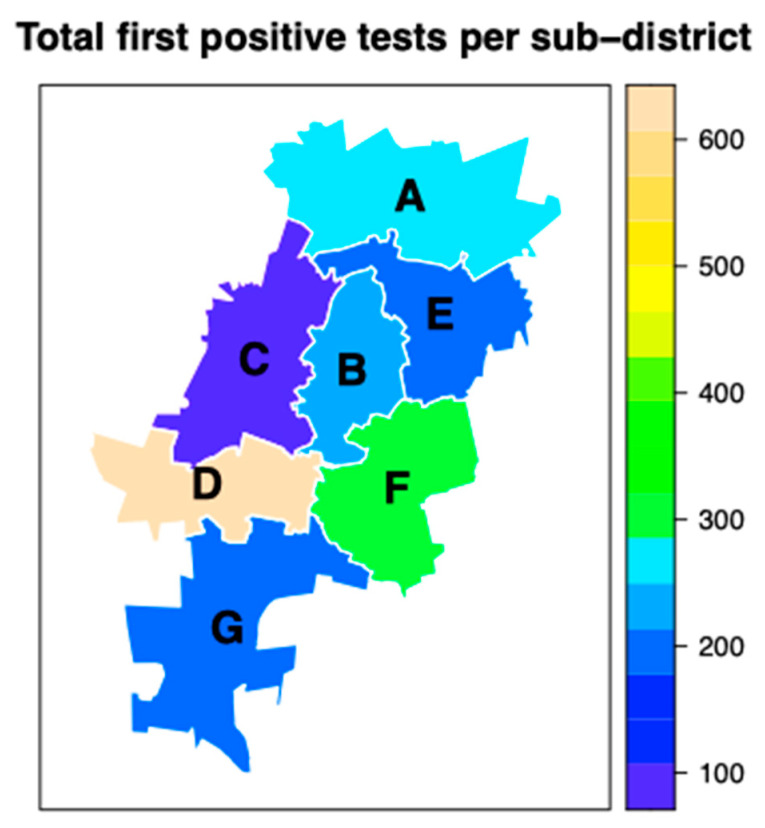
Total first HIV PCR positive tests per subdistrict in the Johannesburg Health District, October 2018–September 2021.

A third of all HIV PCR tests performed in the Johannesburg Health District is performed in subdistrict D (*n* = 46,896), with the least by subdistrict C (*n* = 9 831), a reflection of where the large delivery units, including hospitals, are situated (Figure 3). Unsurprisingly, subdistrict D also yields the first HIV PCR-positive results at 608 over the study period, followed by subdistrict F (*n* = 301) and subdistrict A (*n* = 279).

## 4. Discussion

In this 3-year review of HIV PCR data of children less than two years of age tested in the Johannesburg Health District, there was an increase in tests performed across all age groups. The increase was more pronounced in testing around six months of age, where there was a three-fold increase in HIV PCR testing during the study period, in line with revised PMTCT guidelines. There was an overall decrease in positive HIV PCR tests, translating to a declining annual case rate of newly diagnosed children less than two years of age. The period associated with stringent COVID-19 regulations early in the pandemic in South Africa showed a decrease in positive HIV PCR tests identified with birth and 10-week testing, with a subsequent rebound when restrictions were eased. The majority (79%) of first HIV PCR-positive tests were detected in the first eight months of life.

The finding of minimal interruptions to infant HIV testing during the peak of the COVID-19 pandemic and associated restrictions in South Africa, and interruptions in health services, is reassuring and shows the robustness of the PMTCT program in the Johannesburg Health District. This finding is similar to that of a retrospective review of routinely collected data in KwaZulu-Natal province, South Africa, where there was evidence of a significant decline in birth PCR testing in the period March to May 2020—the period coinciding with the most restrictive COVID-19 regulations—with recovery in testing rates seen in June 2020 [16]. While there was no obvious decrease in the total number of PCR tests performed during the stringent lockdown period in our study, the rebound in positive HIV PCR tests following the easing of restrictions was evident for birth (0–<6 weeks) testing. This is likely to represent a combination of increased and duplicate testing across different facilities where there would have been concerns of reduced testing and/or less access to laboratory records to check results. The algorithm deduplicating laboratory test records perform less accurately across different facilities. In another review of programmatic data from 65 primary care facilities also in KwaZulu Natal, there was an almost 50% decline in HIV testing and ART initiation during the early stages of the COVID-19 lockdown [17]. There were, however, no details on early infant diagnosis. 

The increase in testing at around six months of age reflects the successful implementation of a component of the revised 2019 PMTCT guidelines. This is reassuring given the reported interruptions in HIV care with the COVID-19 pandemic and the 2020 UNICEF report that showed the limited implementation of the revised guidelines [8]. While our study did not assess dolutegravir use in PWLHIV and interruptions in accessing ART during the lockdowns, the decrease in positive HIV PCR tests in children less than two years of age could be attributable to the transition to dolutegravir-based first-line ART regimens. Dolutegravir-based ART has been shown to achieve a faster rate of viral load suppression compared to non-nucleoside reverse transcriptase-based ART [18]. While there was a decrease in cases of children with HIV, the decrease might be smaller than anticipated with dolutegravir-based ART due to interruptions in accessing ART. 

In our study, 79% of first HIV PCR-positive tests were identified in infants aged eight months or less, a finding similar to that reported by Goga et al. [14]. In their review of a nationally representative sample of 9120 HIV-exposed infants, 81% of cumulative MTCT cases had occurred by six months. While there was a good correlation in total HIV PCR tests performed between the NICD and DHIS data in our study, there was a large disparity in the number of first HIV PCR-positive tests for birth and 10-weeks testing. This has important implications as DHIS data are used for modelling, planning and tracking progress toward eMTCT. The data are also important in ensuring that children that test HIV positive are linked to care. The spatial analysis in our study on where the HIV PCR-positive cases are is important for the allocation of resources for PMTCT and HIV care for children who test positive.

While the large NICD infant HIV PCR testing database is a strength of our study, there are several limitations. The absence of a unique patient identifier and thus eliminating duplication of tests is an important limitation. The NICD DW probabilistic linking algorithm, while intended to minimise this duplication, is not without limitations as it relies on demographic data provided by clinicians. The mobility of patients may also contribute to the duplication of tests. Despite the limitations, our study contributes important data on the robustness of an established PMTCT programme and how there were minimal interruptions in the implementation of guidelines despite the devastating impact of COVID-19 on health services. While there has been a decline in the number of children under the age of two years testing HIV PCR positive, much work remains in ensuring that the number decreases further as the case rates in our study are far above what is required for eMTCT, which is <50 HIV PCR positive cases per 100 000 live births [19,20]. We also need to ensure that those children who test positive are linked to and remain in care. 

## Figures and Tables

**Table 1:** Comparison of NICD and DHIS HIV PCR tests performed and positive tests identified, October 2018–September 2021.

	NICD Data						DHIS			
	<2 Years		Birth		10 Weeks		Birth		10 Weeks	
Year	Total Tests	Positive Tests	Total Tests	Positive Tests	Total Tests	Positive Tests	Total Tests	Positive Tests	Total Tests	Positive Tests
Year 1	41 879	659	18 254	206	15 478	189	16 884	98	14 997	76
Year 2	47 265	640	19 869	236	15 738	157	17 639	108	16 137	108
Year 3	56 813	620	20 533	189	17 766	144	17 292	120	19 201	80
Total	145 957	1 919	58 656	631	48 982	490	51 815	326	50 335	264

NICD = National Institute for Communicable Disease; DHIS = District Health Information System; Year 1 = 1 October 2018 to 30 September 2019; Year 2 = 01 October 2019 to 30 September 2020; Year 3 = 01 October 2020 to 30 September 2021.

## Data Availability

De-identified data available on request.
